# Supplementary material for: EF-P and its paralog EfpL (YeiP) differentially control translation of proline-containing sequences
Source: Nat Commun. 2024 Dec 2;15:10465. doi: 10.1038/s41467-024-54556-9 (PMC11611912; doi:10.1038/s41467-024-54556-9)
Supplement: Supplementary file 1 — Supplementary Information [file 41467_2024_54556_MOESM1_ESM.pdf]

SUPPLEMENTARY INFORMATION

EF-P and its paralog EfpL (YeiP) differentially control translation of proline-containing sequences

Alina Sieber[1], Marina Parr[2]*, Julian von Ehr[3,4], Karthikeyan Dhamotharan[3], Pavel Kielkowski[5], Tess Brewer[1], Anna Schäpers[1], Ralph Krafczyk[1], Fei Qi[6], Andreas Schlundt[3,7], Dmitrij Frishman[2] & Jürgen Lassak[1+]*

[1]Faculty of Biology, Microbiology, Ludwig-Maximilians-Universität München, 82152 Planegg-Martinsried, Germany
[2]Department of Bioinformatics, Wissenschaftzentrum Weihenstephan, Technische Universität München, 85354 Freising, Germany
[3]Institute for Molecular Biosciences and Biomolecular Resonance Center (BMRZ), Goethe University Frankfurt, Max-von-Laue-Str. 7-9, 60438 Frankfurt, Germany.
[4]IMPRS on Cellular Biophysics, Max-von-Laue-Str. 7-9, 60438 Frankfurt, Germany.
[5]Department of Chemistry, Institut für Chemische Epigenetik (ICEM), Ludwig-Maximilians-Universität München, 81377 Munich, Germany
[6]State Key Laboratory of Cellular Stress Biology, School of Life Sciences, Xiamen University, 361102 Xiamen, China
[7]University of Greifswald, Institute of Biochemistry, Felix-Hausdorff-Str. 4, 17489 Greifswald, Germany

*These authors contribute equally; [+]corresponding author: juergen.lassak@lmu.de

A

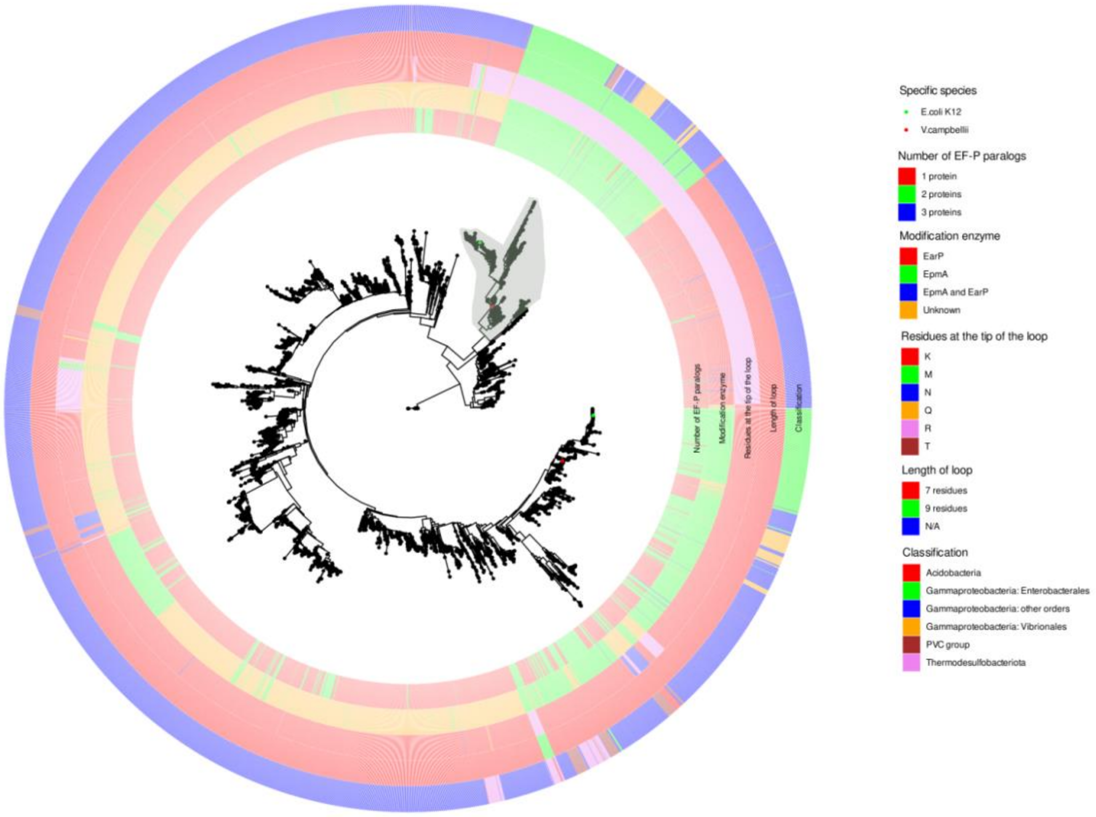

B

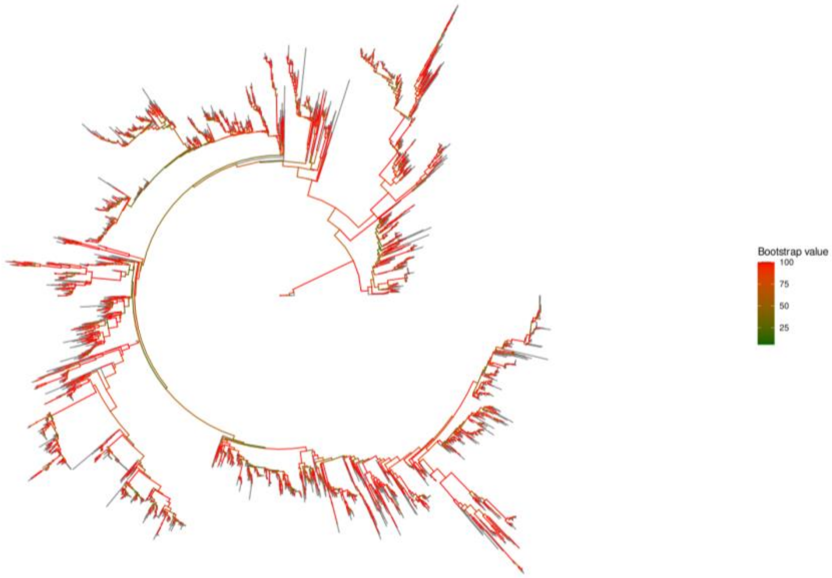

Supplementary Fig. 1: Phylogenetic analysis of EF-P subgroups

Phylogenetic tree was built using the multiply aligned 5448 sequences of KOW-like domains of proteins that have three domains typical for EF-P in a collection of 4736 complete bacterial genomes was obtained from the RefSeq database[25]. **(A)** Outer ring shows phylogenetic classification in bacterial phyla. Other rings show tip residues and length of β3Ωβ4 loop, as well as number of EF-P homologs and modification enzymes found in bacterial proteomes. Branch endings indicate affiliation to specific species. The green highlighting indicates the branch with protein sequences further annotated as EfpL proteins. **(B)** Corresponding bootstrap values.

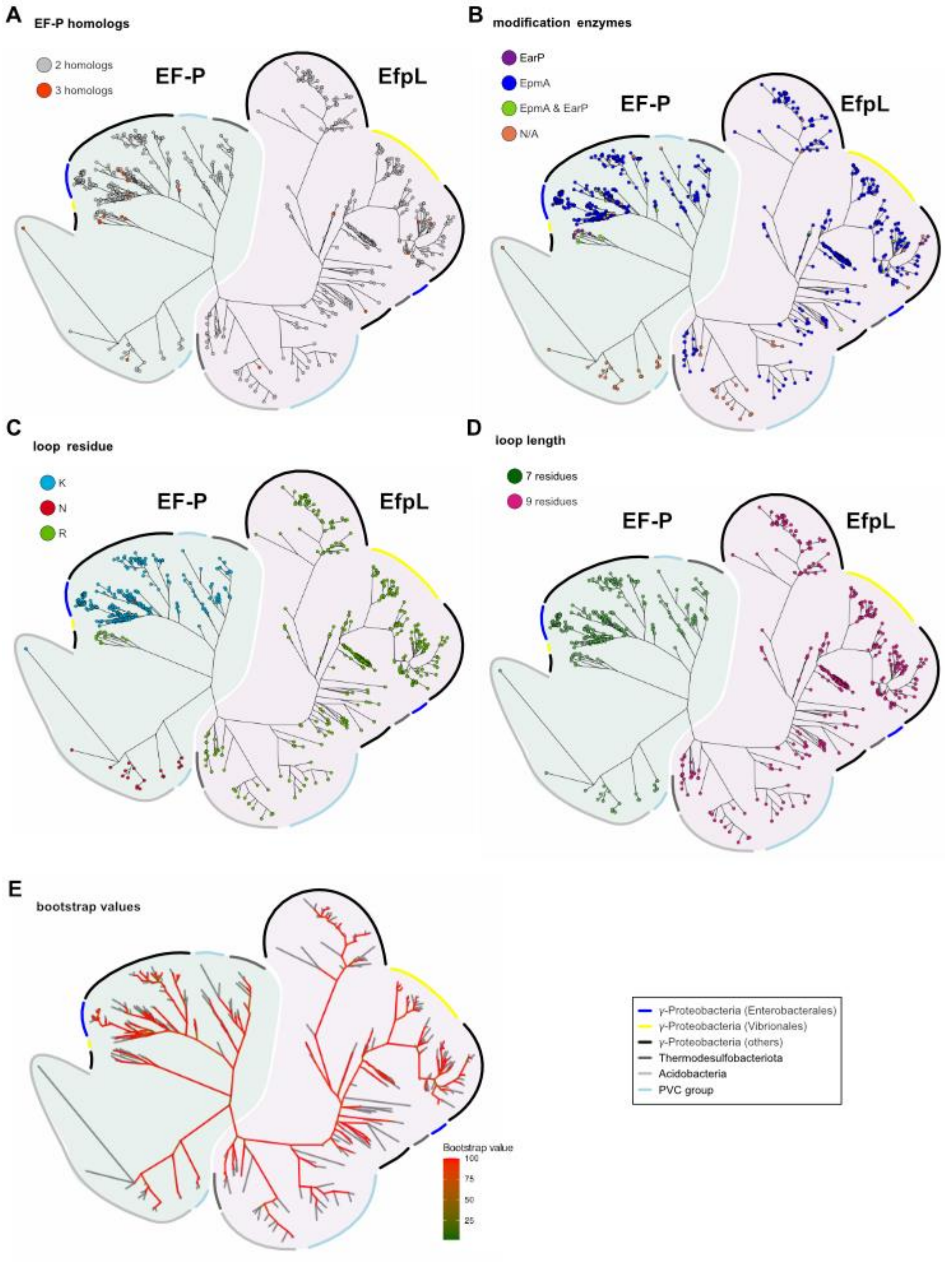

Supplementary Fig. 2: Phylogenetic analysis of co-occurring EF-P and EfpL proteins

Phylogenetic tree of EfpL (purple) and co-occurring EF-Ps (green). Colored lines indicate bacterial phyla. **(A)** EF-P homologs per proteome. **(B)** EF-P modification enzymes found per proteome. **(C)** β3Ωβ4 loop tip residue in EF-P or EfpL. **(D)** β3Ωβ4 loop length of EF-P or EfpL. **(E)** Corresponding bootstrap values.

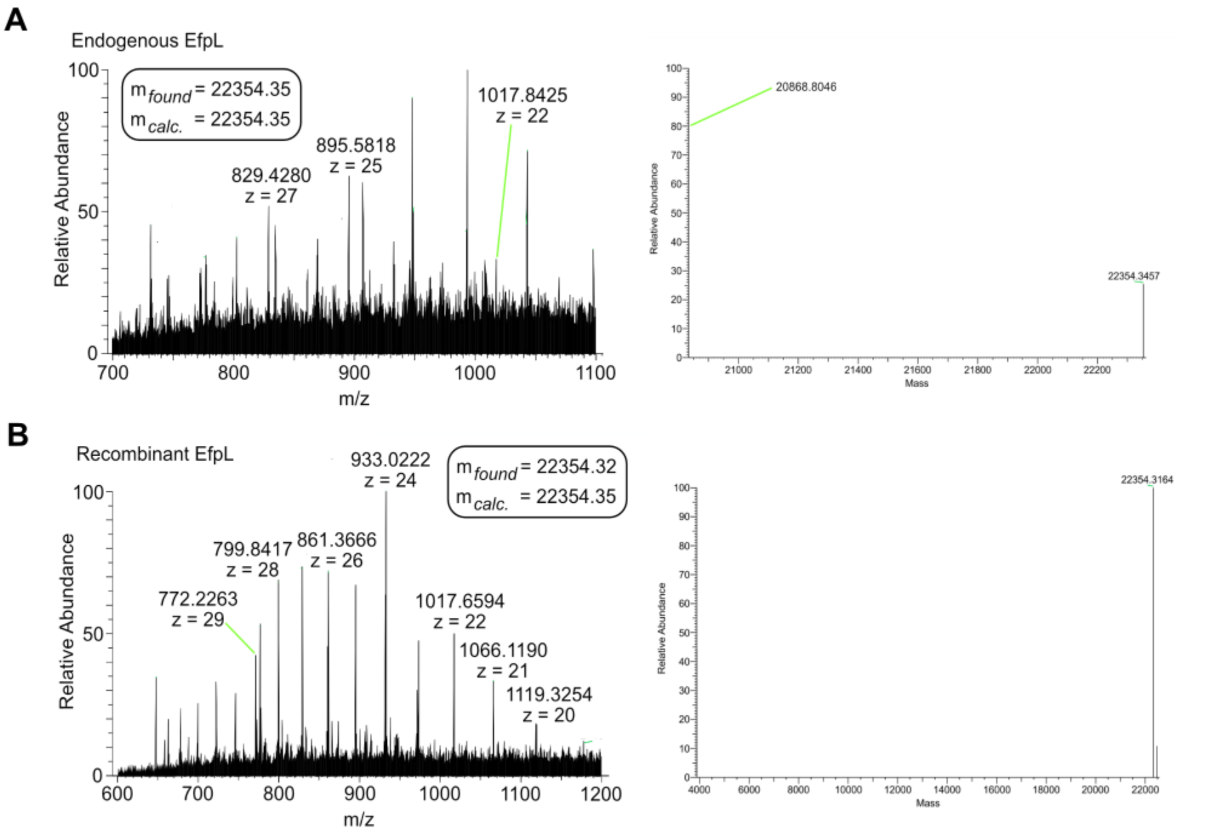

Supplementary Fig. 3: Mass spectrometry (MS) for EfpL protein analysis

MS spectra for **(A)** endogeneous *E. coli* EfpL (n=1, multiple spectra from same protein sample) and **(B)** recombinant produced *E. coli* EfpL (n=1, multiple spectra from same protein sample) to identify modification status. Left side: DDA raw files; right side: output mzML format. Mass ($m_{calc.}$) was calculated according the Uniprot database (identifier: B7UFI8 - EFPL_ECO27) [1]. At least two unique peptides were required for protein identification. False discovery rate determination was carried out using a decoy database and thresholds were set to 1 % FDR.

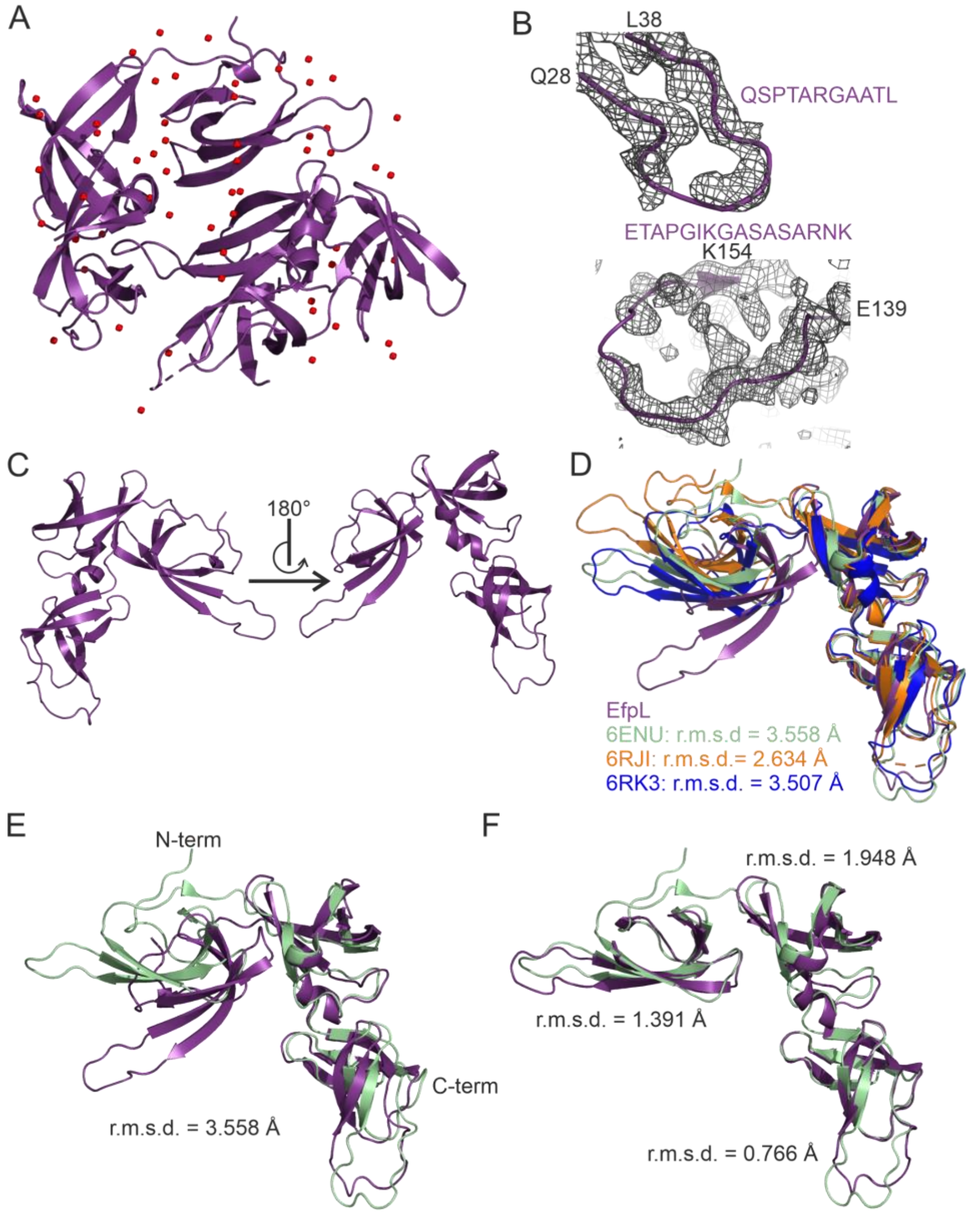

Supplementary Fig. 4: Structural comparison of EfpL and EF-P

(A) Structure of crystallographic dimer of EfpL determined by x-ray crystallography in this study. Waters and co-crystallized glycerol ligands are colored in red and blue respectively. **(B)** Electron density map of KOW β3Ωβ4 loop (upper panel) and OB d3 loop 1 (lower panel). The 2Fo-Fc electron density is contoured at 1.5 σ. **(C)** Two-sided view of fully build single chain from the EfpL x-ray structure in A. **(D)** Structural alignment of EfpL with EF-P from *E. coli* (cryo-EM structure, PDB entry 6ENU) and *S. aureus* (crystal structure, PDB entry 6RJI and NMR solution structure, PDB entry 6RK3). R.m.s.d. values in comparison to EfpL are shown. **(E)** Structural alignment of EfpL with EF-P from *E. coli* (cryo-EM structure, PDB entry 6ENU). Root mean square deviation (r.m.s.d) of the total alignment is shown. **(F)** The same as in E but with structured domains (residues 4-56, 68-128, 132-187) of EfpL separated and aligned individually to EF-P and are shown with respective r.m.s.d.

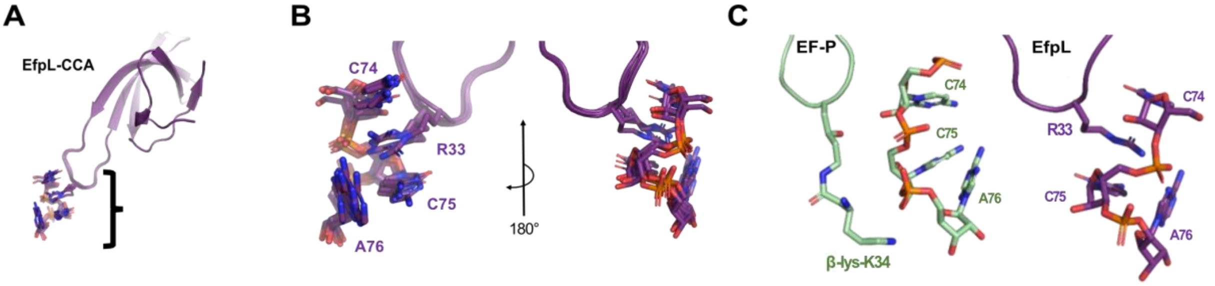

Supplementary Fig. 5: *In silico* interaction analysis of EF-P and EfpL with the tRNA CCA trinucloetide with the RNA trinucleotide

(A) Full-view superimposition of the four best solutions obtained from a HADDOCK run of EfpL together with $_{74}$CCA$_{76}$. **(B)** Zoom-in of panel A to the KOW domain β3Ωβ4 loop region in contact with the RNA trinucleotide. The r.m.s.d. is 0.12 ± 0.01 Å. **(C)** Excerpt of β3Ωβ4 loops from EF-P and EfpL (see panels A and B) in complex with the tRNA trinucleotide CCA. The central tip residue of EF-P is β-lysylated and the depicted complex based on PDB ID 6ENU[3].

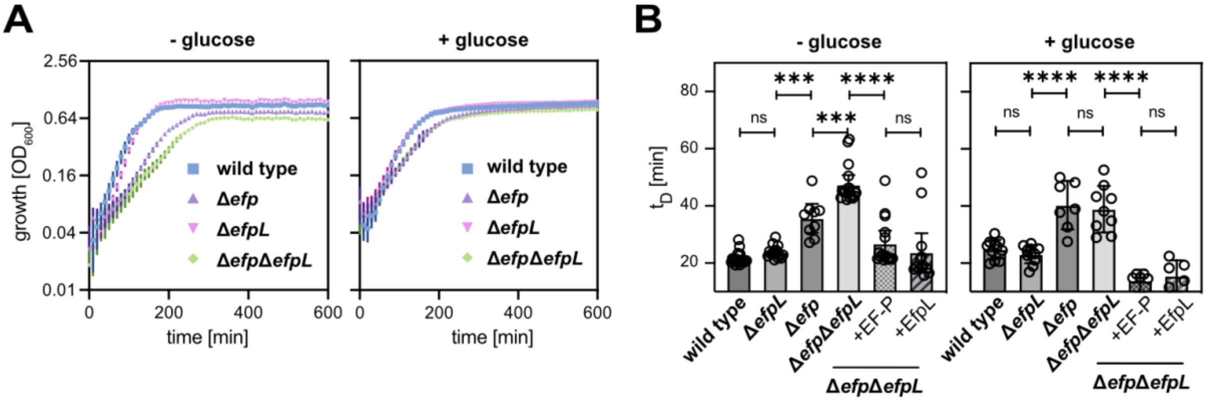

Supplementary Fig. 6: Growth analysis of *E. coli* wild type and deletion strains.

Growth analysis of *E. coli* BW25113 wild type and deletion strains in LB with and without addition of 20 mM glucose. **(A)** Growth curves shown for 600 minutes (n=10, biological replicates, mean with sd indicated as error bars). **(B)** For complementation *efp* (+EF-P) or *efpL* (+EfpL) were provided in trans. Doubling times (t_D) were calculated from exponentially grown cells in LB (n ≥ 6, biological replicates, mean with sd indicated as error bars) Statistically significant differences according to ordinary one-way ANOVA test with multiple comparison (*P value <0.0332, **P value <0.0021, ***P value <0.0002, ****P value <0.0001, ns not significant). **(A&B)** Source data are provided as a Source Data file

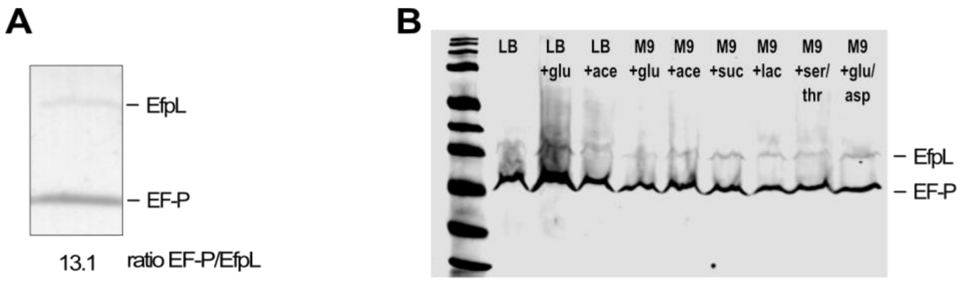

Supplementary Fig. 7: Protein amount of EF-P and EfpL in *E. coli*

To quantify endogenous production of EF-P and EfpL a 6xHis encoding sequence was genomically integrated at the 3' end of the ORFs of *efp* and *efpL* in *E. coli* BW25113. **(A)** Production in LB was quantified via immunoblotting using Anti-His6 antibodies. Ratio determined using Fiji[2]. **(B)** Potein production was detected with different growth media, LB and LB supplemented with 40 mM glucose (LB+glu), and minimal media with different C-sources: M9 with 40 mM glucose (M9+glu), 40 mM acetate (M9+ace), 40 mM succinate (M9+suc), 40 mM lactose (M9+lac), 20mM serine together with 20 mM threonine (M9+ser/thr), and 20 mM glutamate together with 20 mM aspartate (M9+glu/asp). **(A&B)** Source data are provided as a Source Data file

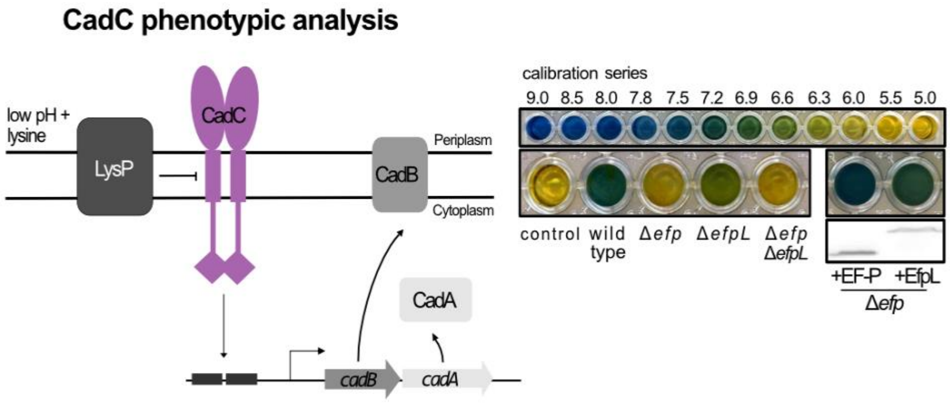

Supplementary Fig. 8: CadC phenotypic analysis

Scheme of CadC dependent pH regulation[3]: To visualize pH regulation, cells were cultivated in lysine decarboxylase indicator medium (indicator: bromothymol blue) and alkalization is depicted as a color change from yellow over green to blue (n=1). Production of EF-P and EfpL was confirmed by immunodetection of the C-terminally attached His6-tag using α-His$_6$ antibody. Source data are provided as a Source Data file.

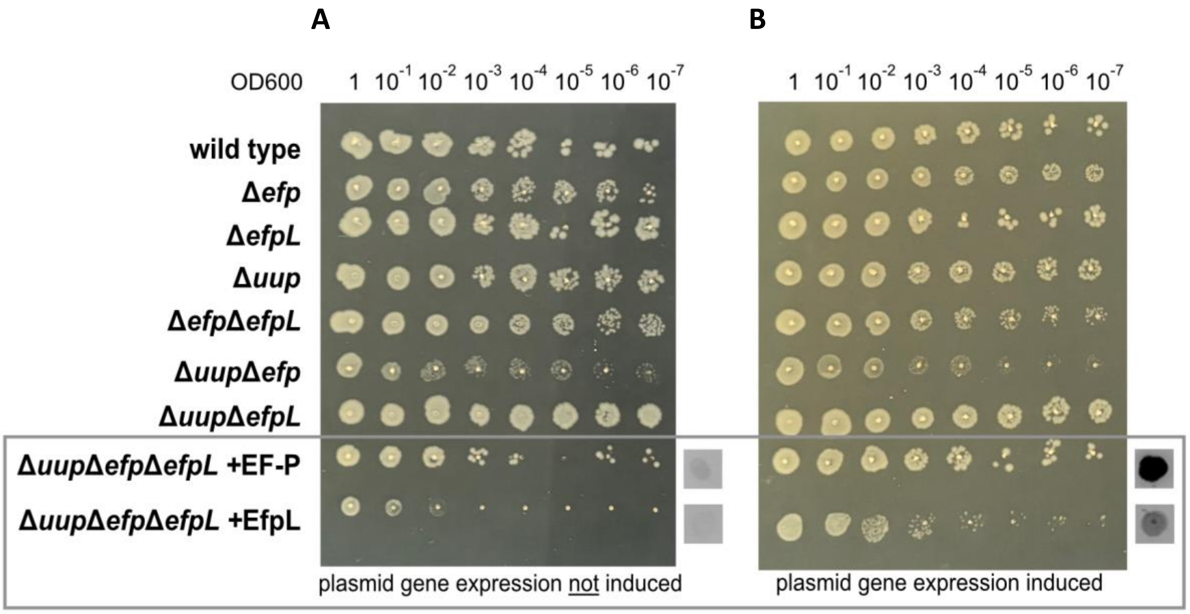

Supplementary Fig. 9: Growth analysis of *E. coli* wild type and mutant strains on plate

E. coli BW25113 wild type and mutants were grown over night in liquid media and spotted in different dilutions on LB plates containing **(A)** 40 mM glucose or **(B)** 40 mM arabinose, respectively. Expression status of complementation of Δ*uup*Δ*efp*Δ*efpL* with EF-P or EfpL on an arabinose inducible promoter was checked via α-His_6 antibody on a dot blot. **(A&B)** Source data are provided as a Source Data file

A

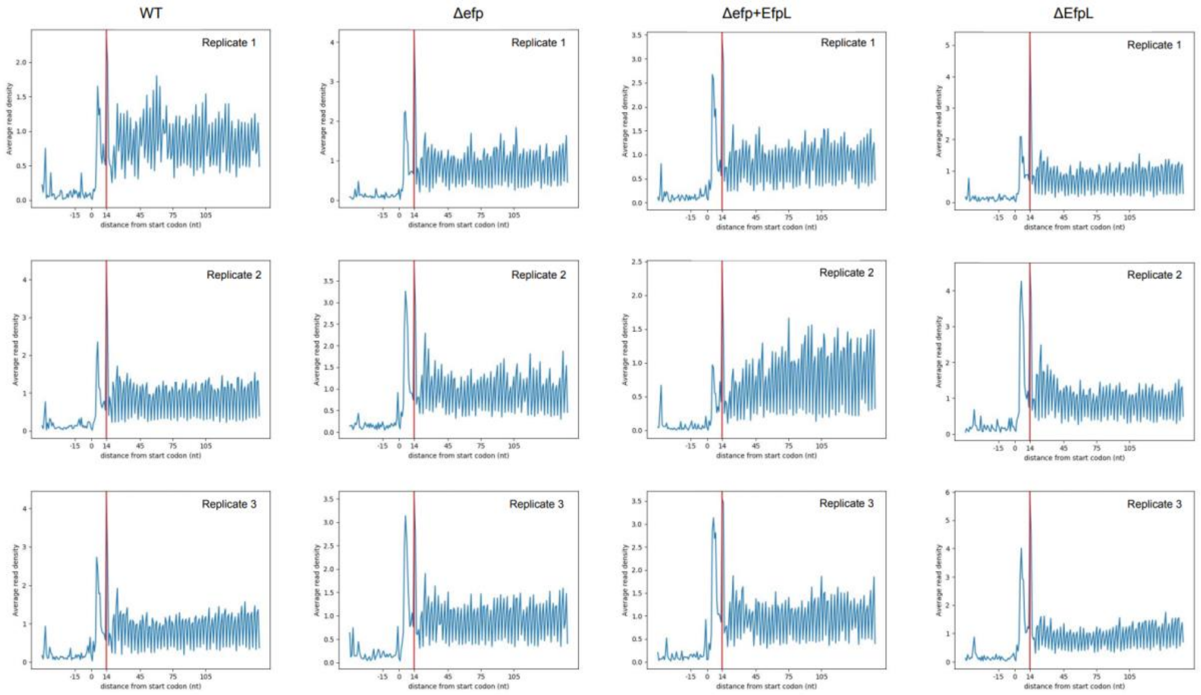

B

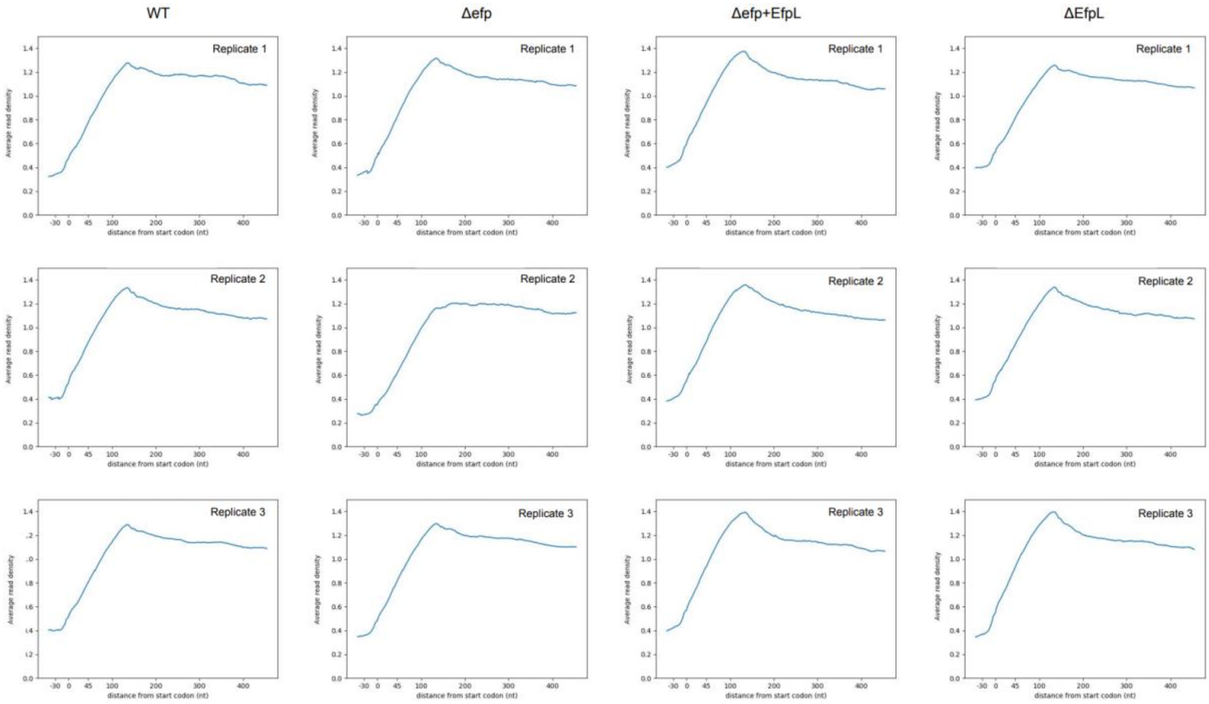

Supplementary Fig. 10: Metagene plots in the region of initiation

Metagene plots for Ribo-seq and RNA-seq samples of the wildtype (WT), *efp* deletion mutant (Δ*efp*), *efp* deletion mutant with overexpression of *efpL* (Δ*efp*+EfpL), and *efpL* deletion mutant (Δ*efpL*). The x-axis shows **(A)** the distance from the footprint or **(B)** RNA-seq read to the start or stop codons; the y-axis represents the average read density of the position. In the metagene plots for Ribo-seq samples (A), the 3'-end of the read was used to indicate the location of each footprint.

A

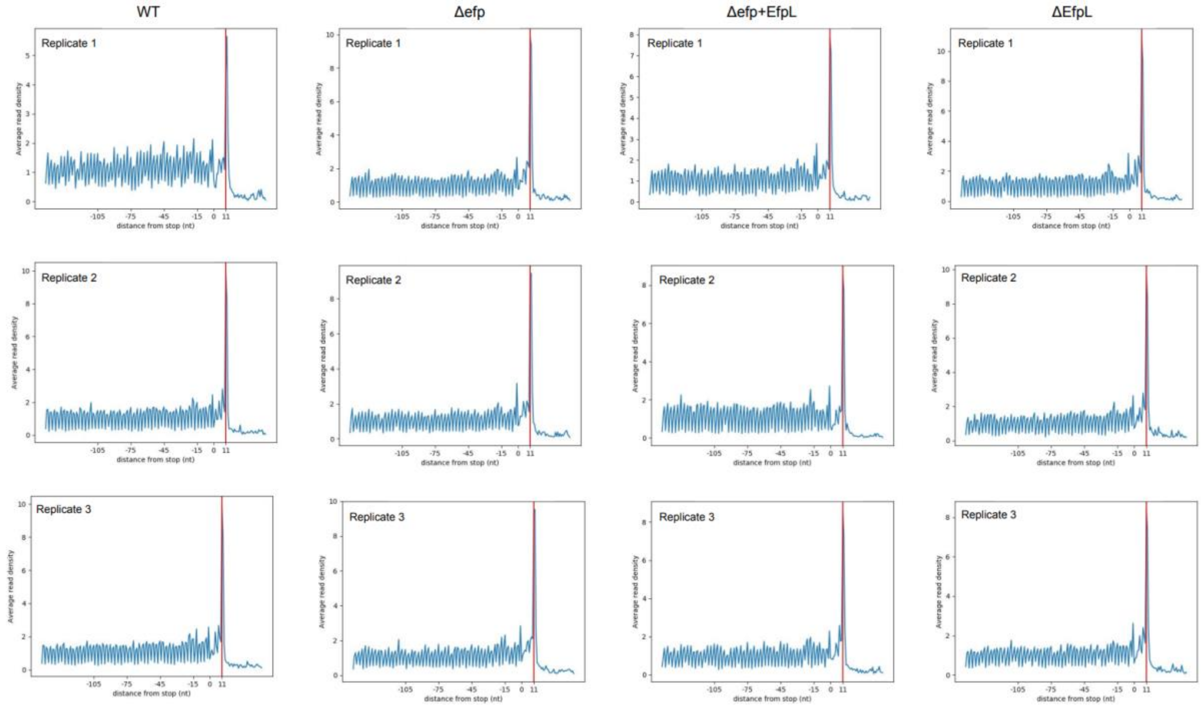

B

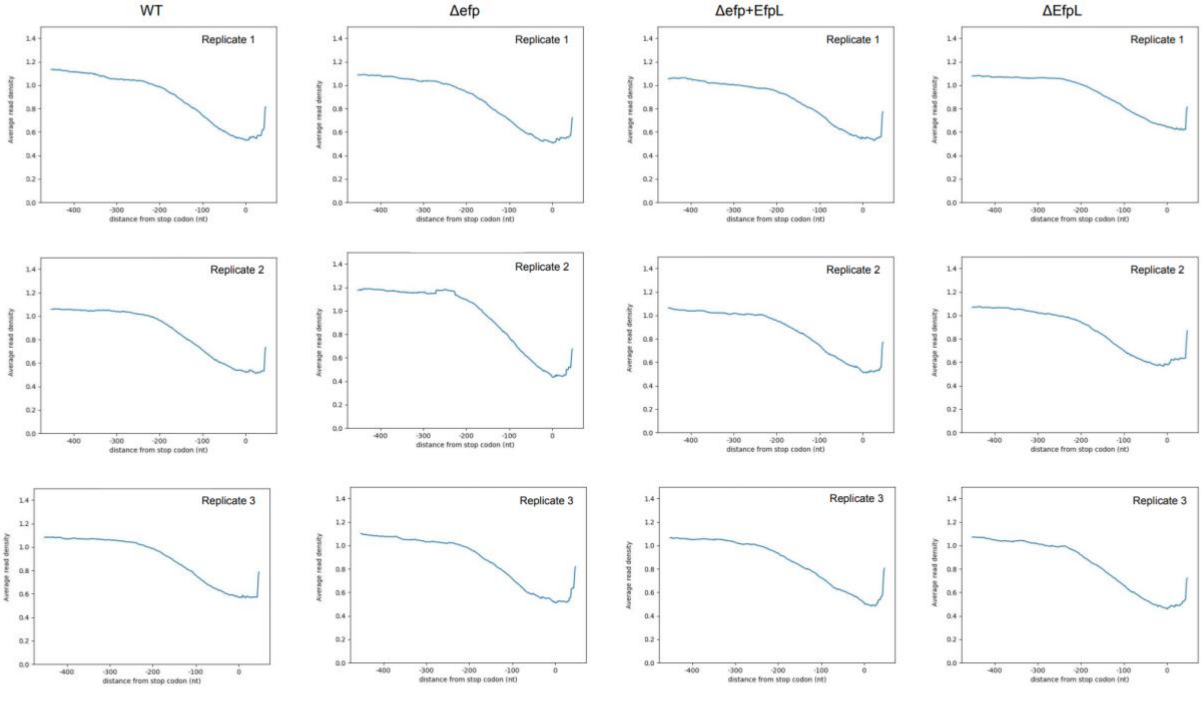

Supplementary Fig. 11: Metagene plots in the region of termination

Metagene plots for Ribo-seq and RNA-seq samples of the wildtype (WT), *efp* deletion mutant (Δ*efp*), *efp* deletion mutant with overexpression of *efpL* (Δ*efp*+EfpL), and *efpL* deletion mutant (Δ*efpL*). The x-axis shows **(A)** the distance from the footprint or **(B)** RNA-seq read to the start or stop codons; the y-axis represents the average read density of the position. In the metagene plots for Ribo-seq samples (A), the 3'-end of the read was used to indicate the location of each footprint.

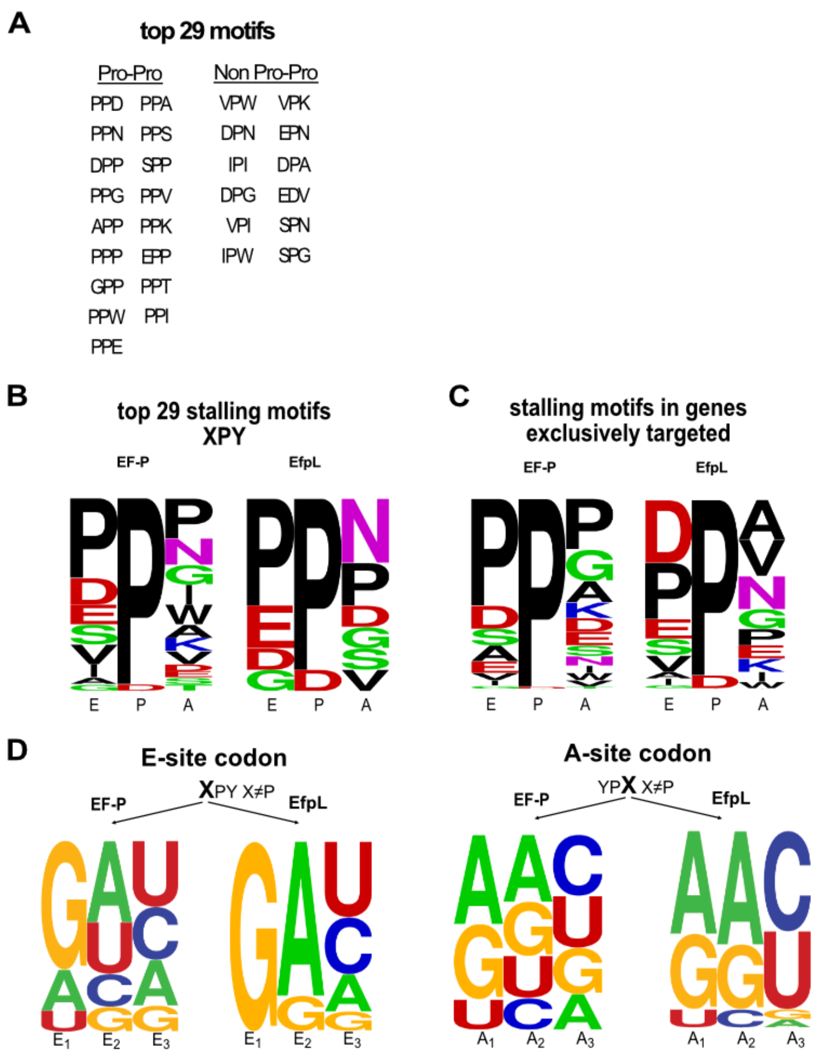

Supplementary Fig. 12: Top 29 motifs in *E. coli* and comparison of codon bias of the E-site and A-site amino acid

(A) Top 29 EF-P dependent arrest motifs associated with ribosome pausing in *E. coli* BW25113 determined by PausePred[36]. **(B)** Sequence logo[26] of the top 29 EF-P dependent arrest motifs. **(C)** Sequence logo[26] of the arrest motifs in genes targeted by exclusively EF-P or EfpL. **(D)** Sequence logos of the E- and A-site codons in XPY or YPX arrest motifs X≠P targeted by EF-P and EfpL, respectively.

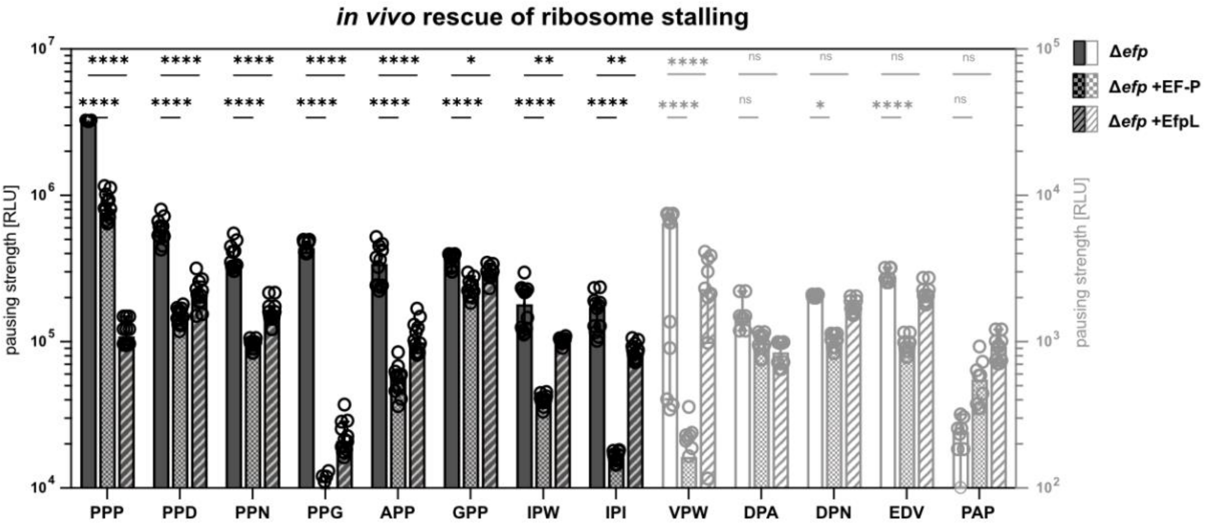

Supplementary Fig. 13: *In vivo* detection of pausing strength at different motifs

In vivo comparison of stalling strength of a set of stalling motifs and negative control PAP of *E. coli* Δ*efp* cells and respective trans complementation with EF-P (Δ*efp* +EF-P) and EfpL (Δ*efp* +EfpL). Pausing strength correlates with light emission and is given in relative light units (RLU) (n = 12, biological replicates, mean with sd indicated as error bars). Statistically significant differences according to 2-way ANOVA test with multiple comparison (*P value <0.0332, **P value <0.0021, ***P value <0.0002, ****P value <0.0001, ns not significant). Source data are provided as a Source Data file

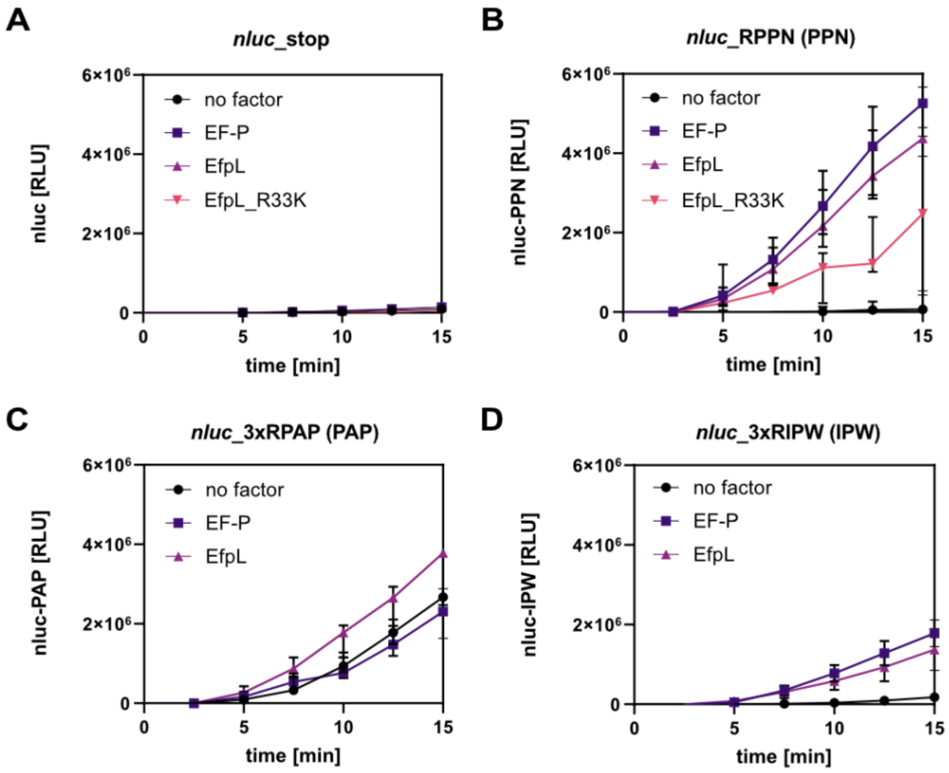

Supplementary Fig. 14: Comparison of EF-P and EfpL of *E. coli* in translating different motifs

In vitro transcription and translation of the *nLuc®* variants **(A)** nLuc_stop, **(B)** nLuc_RPPN (PPN), **(C)** nLuc_3xRIPW (IPW) or **(D)** nLuc_3xRPAP (PAP). The absence (no factor) or presence of the respective translation elongation factors of *E. coli* (EF-P, EfpL) is shown. Translational output was determined by measuring bioluminescence in a time course of 15 minutes (RLU) (n $\geq$ 3, technical replicates, mean with sd indicated as error bars). **(A-D)** Source data are provided as a Source Data file

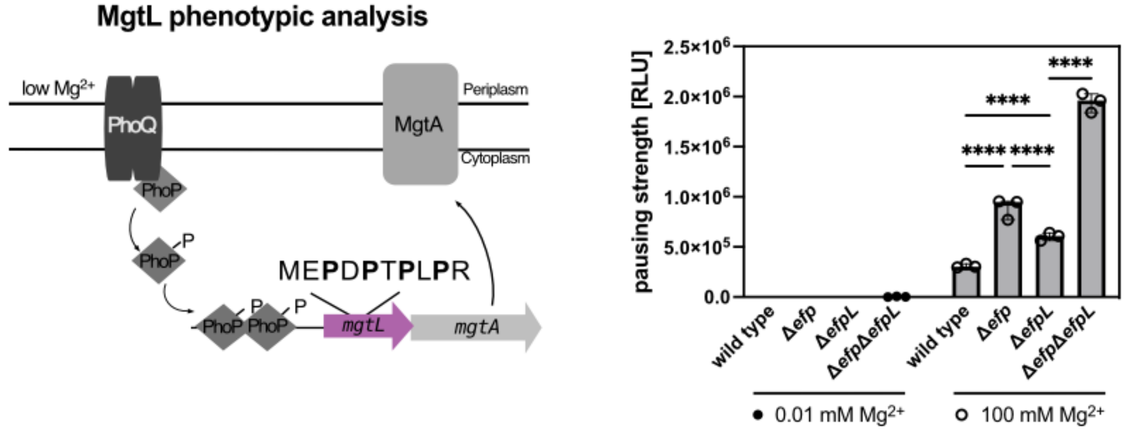

Supplementary Fig. 15: MgtL phenotypic analysis

Left: model illustrating the regulation mechanism of Mg^{2+} uptake by MgtA [4-6]. *mgtL* consists of a proline-rich sequence and regulates *mgtA* expression. Right: reporter assay to detect pausing strength at the MgtL leader peptide with the sequence MEPDPTPLPR. Maximal luminescence emission under high (100 mM) and low (100 µM) Mg^{2+} in *E. coli* BW23113 and corresponding mutant strains is depicted. Pausing strength correlates with light emission and is given in relative light units (RLU). (n = 3, biological replicates, mean with sd indicated as error bars). Statistically significant differences according to two-way ANOVA (*P value <0.0332, **P value <0.0021, ***P value <0.0002, ****P value <0.0001, ns not significant). Source data are provided as a Source Data file.

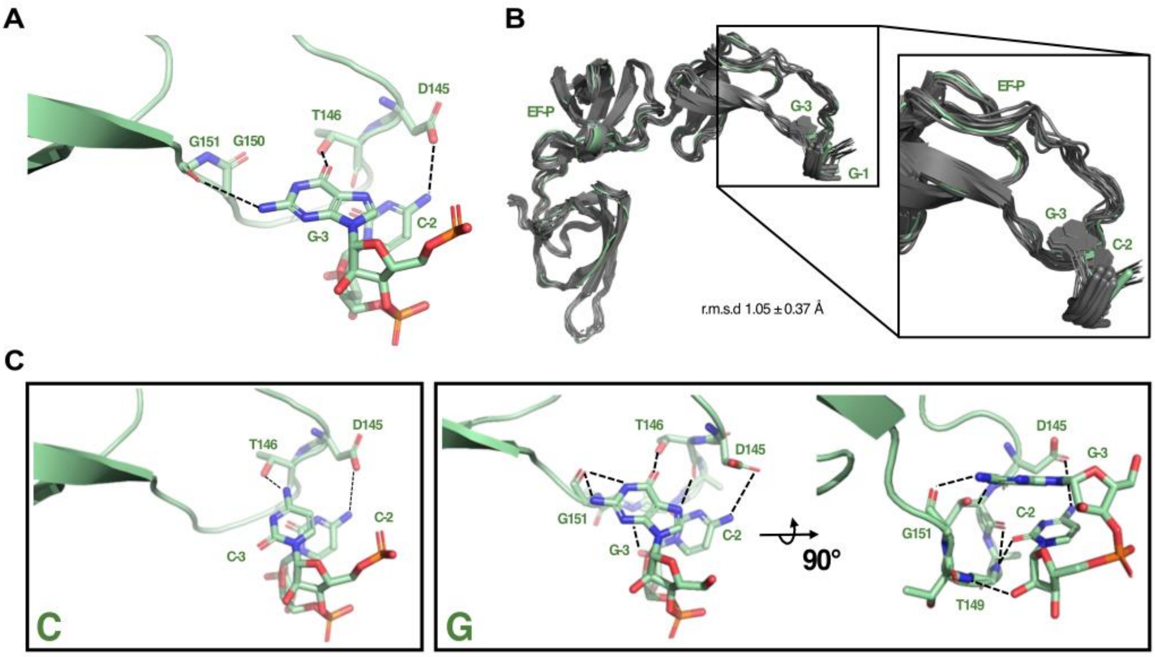

Supplementary Fig. 16: EF-P interaction modelling with E-site codon

(A) Close-up view of EF-P in contact with the E-site codon $_{-3}$GCG$_{-1}$, after *in silico* replacement of $_{-3}$C in the PDB entry 6ENU[7]. Polar contacts to EF-P OB domain 3 loop 1 residues are depicted with broken lines as obtained from the program PyMol (Delano Scientific). Note that only the first two nucleotides are shown for clarity. **(B)** Full-view superimposition of the 10 best solutions obtained from a HADDOCK run of EF-P together with $_{-3}$GCG$_{-1}$. The green model represents the non-docked and non-energy-minimized reference from panel A. The r.m.s.d. is given. The boxed view shows a zoom-in to the EF-P-RNA trinucleotide interface. **(C)** The same as shown in main text Fig. 3, but with an additional perspective depicted for EF-P in complex with GCG to highlight additional contacts.

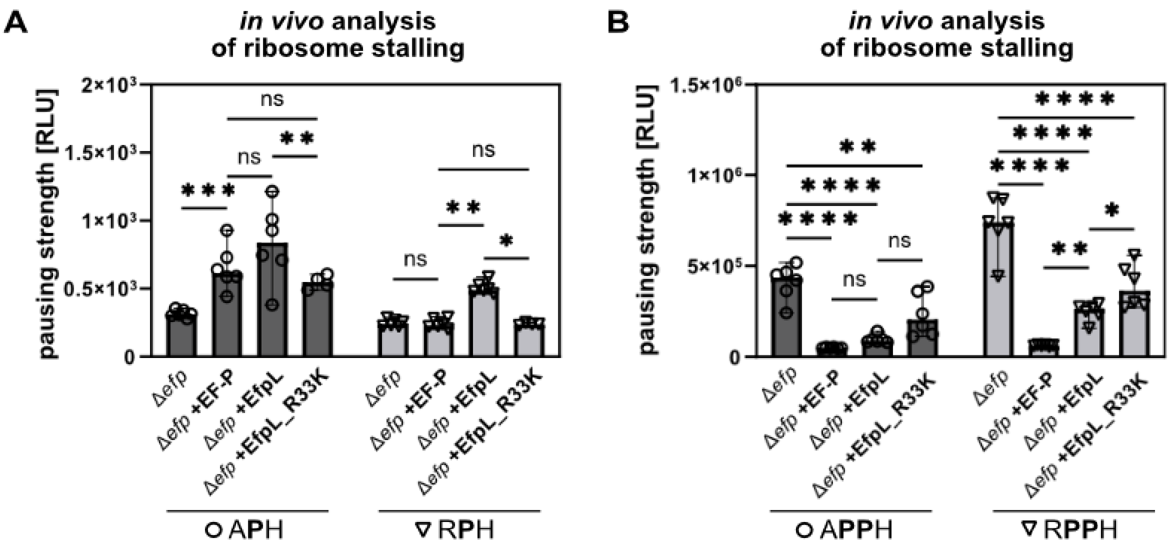

Supplementary Fig. 17: EF-P and EfpL can induce ribosome stalling

In vivo comparison of stalling strength of a **(A)** APH and RPH or **(B)** APPH and RPPH motif in Δ*efp* strains in the absence or presence of *efp* (+EF-P), or *efpL* (+EfpL). Pausing strength correlates with light emission and is given in relative light units (RLU). ($n \geq 4$, biological replicates, mean with sd indicated as error bars). Statistically significant differences according to two-way ANOVA test with multiple comparisons (*P value <0.0332, **P value <0.0021, ***P value <0.0002, ****P value <0.0001, ns not significant). **(A&B)** Source data are provided as a Source Data file

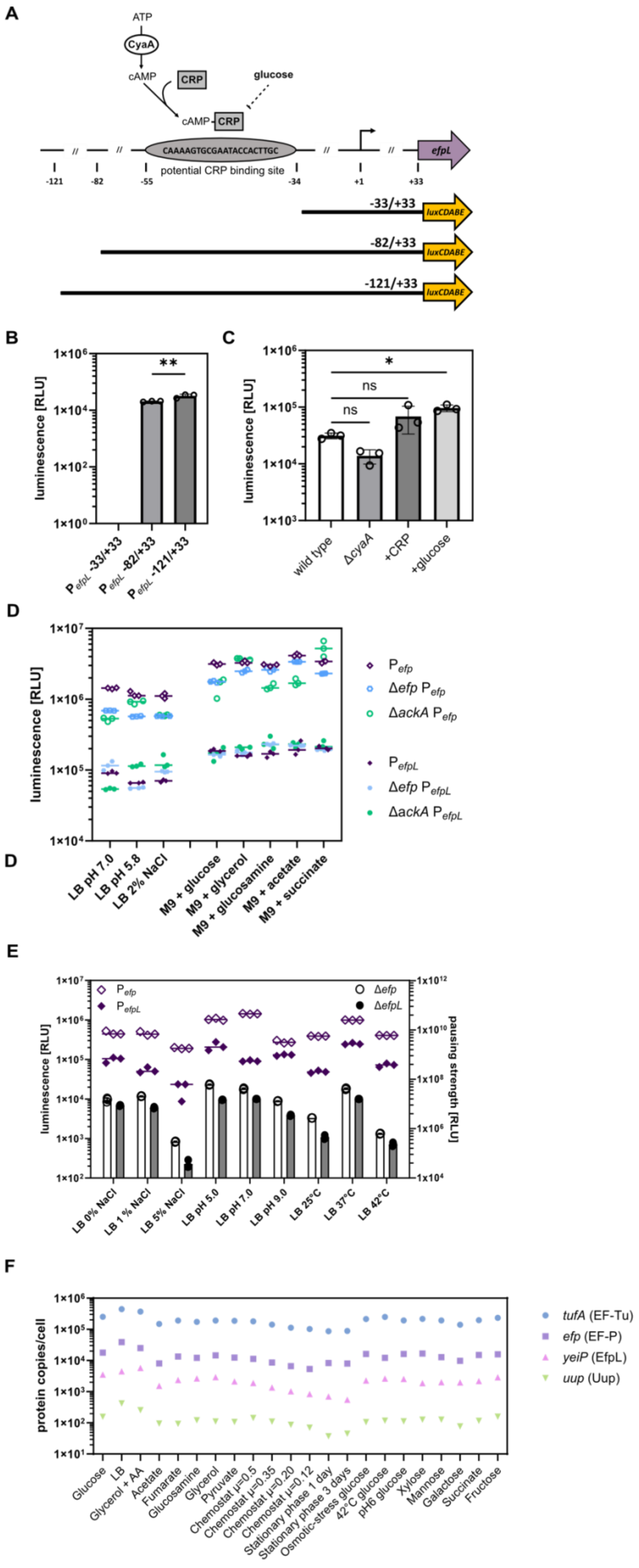
A
ATP
CyaA
cAMP
CRP
glucose
cAMP-CRP
CAAAAGTGCGAATACCACTTGC
efpL
potential CRP binding site
-121
-82
-55
-34
+1
+33
-33/+33
-82/+33
-121/+33
luxCDABE
B
luminescence [RLU]
**
P efpL -33/+33
P efpL -82/+33
P efpL -121/+33
C
luminescence [RLU]
*
ns
ns
wild type
ΔcyaA
+CRP
+glucose
D
luminescence [RLU]
P efp
Δefp P efp
ΔackA P efp
P efpL
Δefp P efpL
ΔackA P efpL
LB pH 7.0
LB pH 5.8
LB 2% NaCl
M9 + glucose
M9 + glycerol
M9 + glucosamine
M9 + acetate
M9 + succinate
E
luminescence [RLU]
pausing strength [RLU]
P efp
P efpL
Δefp
ΔefpL
LB 0% NaCl
LB 1 % NaCl
LB 5% NaCl
LB pH 5.0
LB pH 7.0
LB pH 9.0
LB 25°C
LB 37°C
LB 42°C
F
protein copies/cell
tufA (EF-Tu)
efp (EF-P)
yeiP (EfpL)
uup (Uup)
Glucose
LB
Glycerol + AA
Acetate
Fumarate
Glucosamine
Glycerol
Pyruvate
Chemostat µ=0.5
Chemostat µ=0.35
Chemostat µ=0.20
Chemostat µ=0.12
Stationary phase 1 day
Stationary phase 3 days
Osmotic-stress glucose
42°C glucose
pH6 glucose
Xylose
Mannose
Galactose
Succinate
Fructose

Supplementary Fig. 18: *efp* and *efpL* gene expression analysis

(A) Different lengths of the promoter region or *efpL* (P_{efpL}) were fused with the *luxCDABE* genes of *P. luminescens*. A schematic representation of the catabolite repression with cAMP-CRP shows inhibitory effects of glucose and activating conditions of CyaA for the potential CRP binding site in the promotor region of *efpL*. **(B)** *E. coli* BW25113 wild type strains were transformed with the different promoter reporter fusions. Maximal luminescence of a 16h time course in LB medium is given in relative light units (RLU) (n=3, biological replicates, mean with sd indicated as error bars). Statistically significant differences according to ordinary one-way ANOVA test (*P value <0.0332, **P value <0.0021, ***P value <0.0002, ****P value <0.0001, ns not significant). **(C)** *E. coli* BW25113 strains were transformed with the full-length promoter reporter fusion and tested in LB and LB supplemented with 20 mM CRP or 40 mM glucose. *E. coli* BW25113 Δ*cyaA* strain was transformed with the full-length promoter reporter fusion and tested in LB. Maximal luminescence of a 16h time course in LB medium is given in relative light units (RLU) (n=3, biological replicates, mean with sd indicated as error bars). Statistics as in (B). **(D)** *E. coli* wild type, Δ*efp* or Δ*ackA* strains were transformed with full-length promoter reporter fusion of *efpL* (P_{efpL}) or *efp* (P_{efp}). Maximal luminescence of a 16h time course in LB or M9 minimal medium supplemented with different carbon sources (20 mM) is given in relative light units (RLU) (n=3, biological replicates, line identifies mean value). **(E)** *E. coli* BW25113 strains were transformed with the full-length promoter reporter fusions of *efpL* (P_{efpL}) or *efp* (P_{efp}) and tested in LB and different conditions. Maximal luminescence of a 16h time course in LB medium is given in relative light units (RLU, left y-axis) (n=3, biological replicates, line identifies mean value, purple characters). *In vivo* comparison of stalling strength of Δ*efp* and Δ*efpL* strains for a PPN motif. Pausing strength correlates with light emission and is given in relative light units (RLU, right y-axis) (n=3, biological replicates, mean with sd indicated as error bars, bar chart). **(F)** Quantitative proteome analysis at different conditions for *tufA* (EF-Tu), *efp* (EF-P), *yeiP* (EfpL) and *uup* (Uup) analysed by Schmidt *et al.*[8]. **(A-F)** Source data are provided as a Source Data file

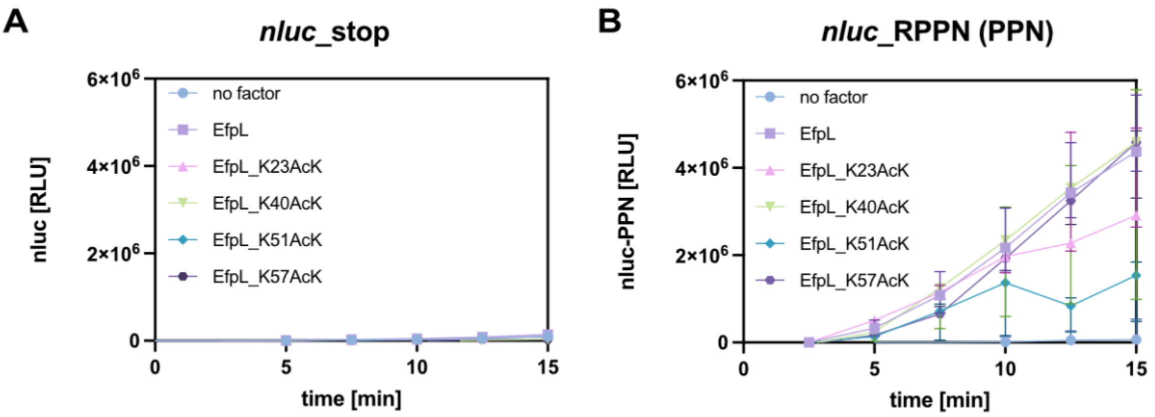

Supplementary Fig. 19: EfpL function dependent on the acylation status

In vitro transcription and translation of the *nLuc®* variants **(A)** nLuc_stop (no motif) or **(B)** nLuc_RPPN (PPN). The absence (no factor) or presence of the respective translation elongation factors of *E. coli* EfpL as well as the corresponding substitution variants EfpL_K23AcK, EfpL_K40AcK, EfpL_51AcK, EfpL_K57AcK is shown. Translational output was determined by measuring bioluminescence in a time course of 15 minutes and is given in relative light units (RLU) (n ≥ 3, mean with sd indicated as error bars). **(A&B)** Source data are provided as a Source Data file

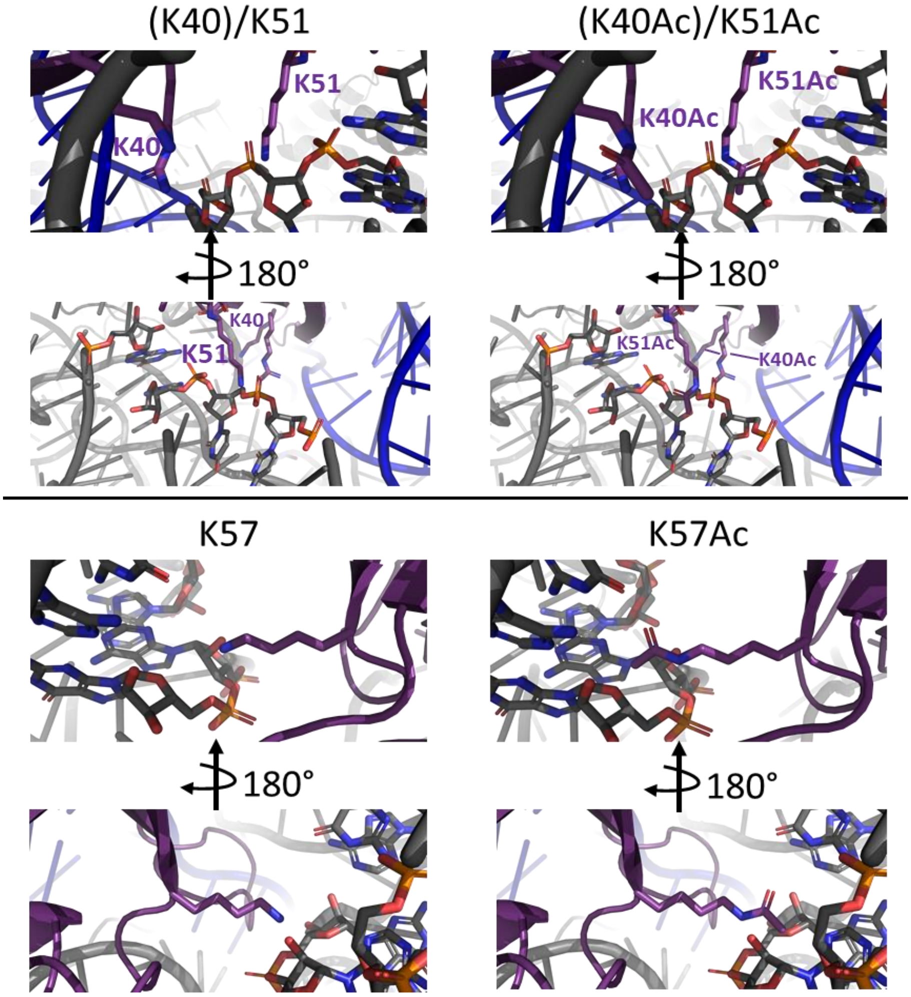

Supplementary Fig. 20: Modelling of Acylation in EfpL

Close-up views on EfpL lysines as shown in unmodified form (left panels) and when acetylated (right panels). Each view is shown from two different perspectives by 180° rotation as indicated. The N-terminal KOW domain (violet-purple) has been aligned to the PDB entry 6ENU[7] to enable monitoring of potential clashes and interactions with ribosomal components. Lysine sidechains are shown as sticks on an otherwise cartoon-typed presentation. For the K40/K51 region (upper panels), R42 is additionally shown to indicate the dense space, relevant in potential sidechain modifications. Relevant RNA regions in close vicinity of lysines are shown as sticks. Grey represents ribosomal RNA, blue indicates tRNA.

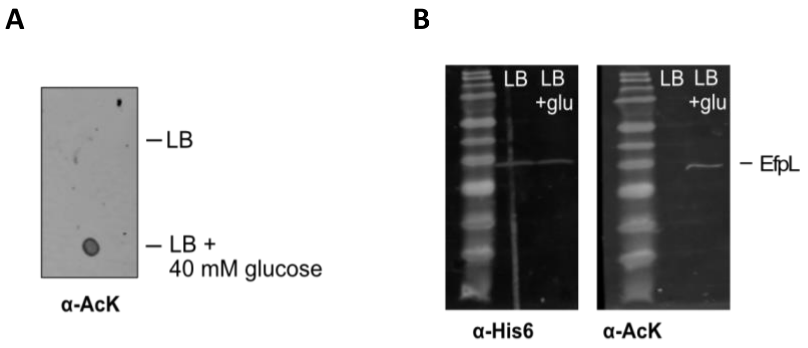

Supplementary Fig. 21: *In vivo* acetylation of EfpL

(A) Immunodetection of lysine acetylation status in BW25113 *efpL*_His grown in LB or LB supplemented with 40 mM glucose. **(B)** Immunodetection of EfpL purified from BW25113 *efpL*_His grown in LB or LB supplemented with 40 mM glucose. **(A&B)** Source data are provided as a Source Data file

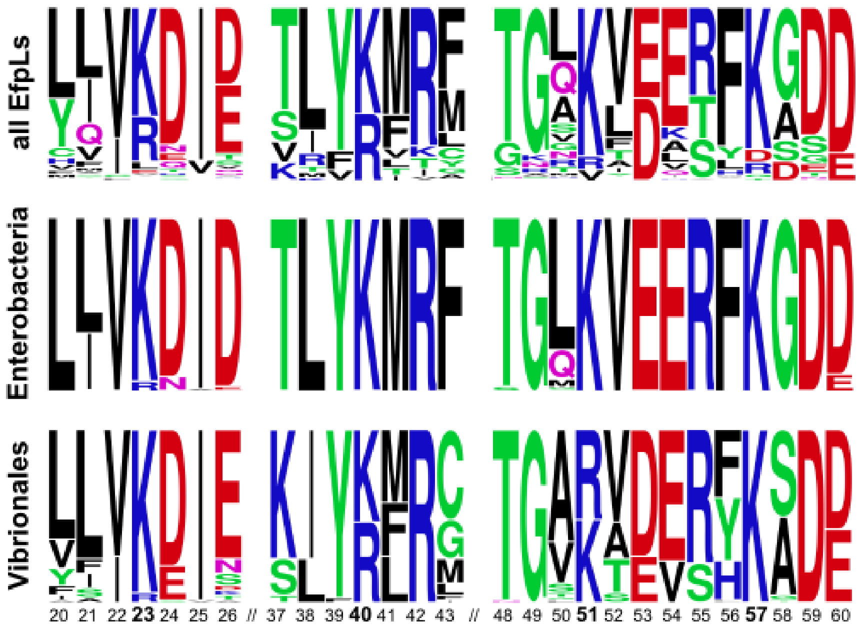

Supplementary Fig. 22: Conservation status of acylation sites

Sequence logos[26] for amino acids at positions 20-60 in all EfpLs, or EfpL from Enterobacteriales or Vibrionales.

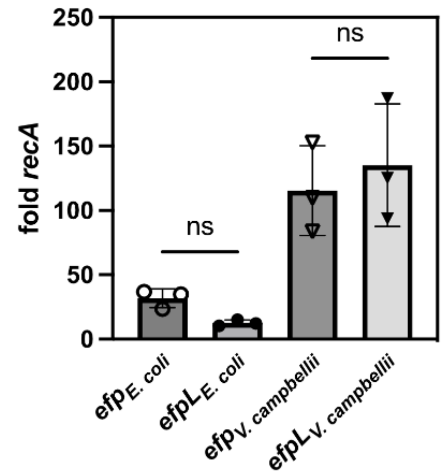

Supplementary Fig. 23: Expression analysis of EF-P and *EfpL* from *E. coli* and *V. campbellii*

Quantitative real time PCR (qRT-PCRs) were performed to analyze expression of *efp* and *efpL* in *E. coli* or *V. campbellii.* (n=3, biological replicates, mean with sd indicated as error bars). Statistically significant differences according to ordinary one-way ANOVA test (*P value <0.0332, **P value <0.0021, ***P value <0.0002, ****P value <0.0001, ns not significant). Primer efficiency was as following: $recA_{E.\ coli}$ 1.987, $efp_{E.\ coli}$ 1.953, $efpL_{E.\ coli}$ 1.936, $recA_{V.\ campbellii}$ 2.084, $efp_{V.\ campbellii}$ 1.962, $efpL_{V.\ campbellii}$ 2.009. Normalization with reference gene *recA* for comparison of *efp* and *efpL* expression. Source data are provided as a Source Data file

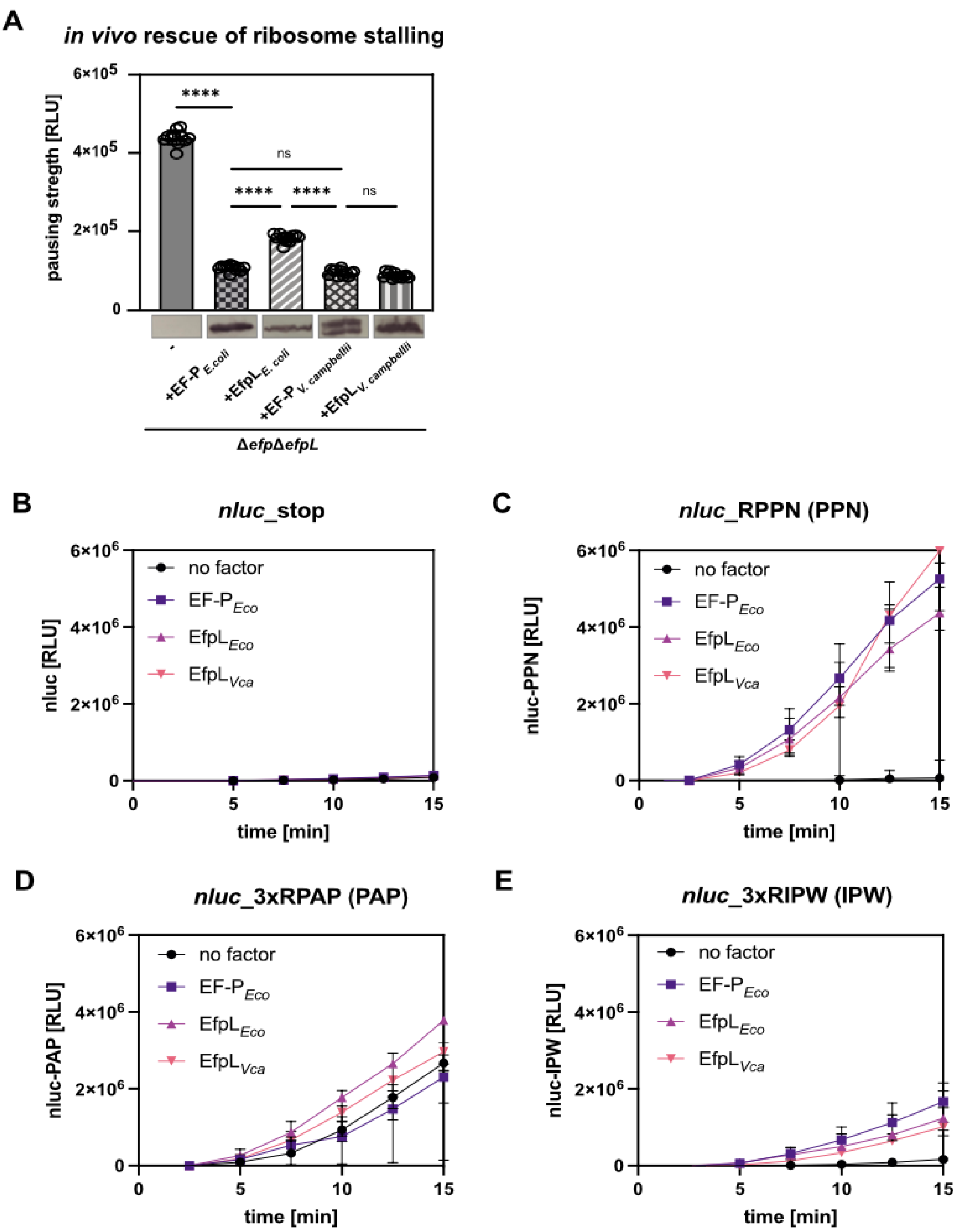

Supplementary Fig. 24: Functional comparison of EfpL from *E. coli* and *V. campbellii*

(A) *In vivo* comparison of stalling strength of a PPN motif of *E. coli* Δ*efp*Δ*efpL* cells and respective trans complementation with *E. coli* EF-P (+EF-P$_{Eco}$) and EfpL (+EfpL$_{Eco}$), as well as *V. campbellii* EF-P (+EF-P$_{Vca}$) and EfpL (+EfpL$_{Vca}$). Production of EF-P and EfpL was confirmed by immunodetection of the C-terminally attached His6-tag using α-His$_6$ antibody. Stalling strength correlates with light emission and is given in relative light units (RLU). (n = 12, biological replicates mean with sd indicated as error bars). Statistically significant differences according to ordinary one-way ANOVA test (*P value <0.0332, **P value <0.0021, ***P value <0.0002, ****P value <0.0001, ns not significant). **(B-E)** *In vitro* transcription and translation of the *nLuc®* variants **(B)** nLuc_stop (no motif), **(C)** nLuc_RPPN (PPN), **(D)** nLuc_3xRIPW (IPW) or **(E)** nLuc_3xRPAP (PAP). The absence (no factor) or presence of the respective translation elongation factors of *E. coli* (EF-P$_{Eco}$, EfpL$_{Eco}$) or *V. campbellii* (EfpL$_{Vca}$) is shown. Translational output was determined by measuring bioluminescence in a time course of 15 minutes (n ≥ 3, technical replicates, mean with sd indicated as error bars). **(A-E)** Source data are provided as a Source Data file

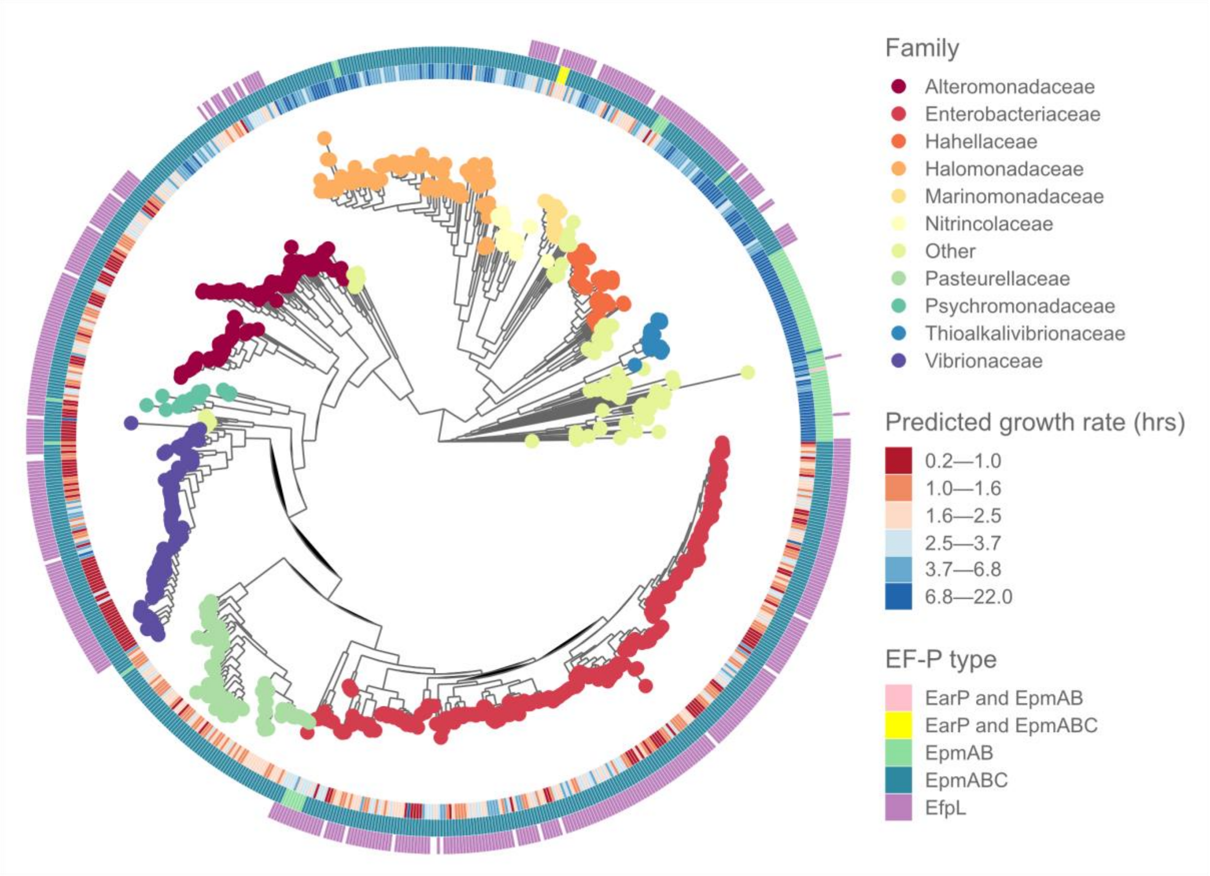

Supplementary Fig. 25: Phylogenetic analysis of predicted growth rates

Set of 920 genomes from the class γ-proteobacteria from the Integrated Microbial Genomes (IMG) database[114]. Inner ring shows doubling times predicted from codon usage bias in ribosomal genes, middle two rings show EF-P types. Colors of tip ends depict phylogenetic family.

Supplementary References

1 Consortium, T. U. UniProt: the Universal Protein Knowledgebase in 2023. *Nucleic Acids Res.* **51**, D523-D531 (2022). https://doi.org:10.1093/nar/gkac1052

2 Schindelin, J. *et al.* Fiji: an open-source platform for biological-image analysis. *Nat. Methods* **9**, 676-682 (2012). https://doi.org:10.1038/nmeth.2019

3 Ude, S. *et al.* Translation elongation factor EF-P alleviates ribosome stalling at polyproline stretches. *Science* **339**, 82-85 (2013). https://doi.org:10.1126/science.1228985

4 Gall, A. R. *et al.* Mg2+ regulates transcription of mgtA in *Salmonella Typhimurium* via translation of proline codons during synthesis of the MgtL peptide. *Proc. Natl. Acad. Sci. U.S.A.* **113**, 15096-15101 (2016). https://doi.org:10.1073/pnas.1612268113

5 Nam, D., Choi, E., Shin, D. & Lee, E. J. $tRNA^{Pro}$-mediated downregulation of elongation factor P is required for mgtCBR expression during *Salmonella* infection. *Mol. Microbiol.* **102**, 221-232 (2016). https://doi.org:10.1111/mmi.13454

6 Takada, H., Fujiwara, K., Atkinson, G. C., Chiba, S. & Hauryliuk, V. Resolution of ribosomal stalling by ABCF ATPases YfmR and YkpA/YbiT. *Nucleic Acids Res.* (2024). https://doi.org/10.1093/nar/gkae556

7 Huter, P. *et al.* Structural Basis for Polyproline-Mediated Ribosome Stalling and Rescue by the Translation Elongation Factor EF-P. *Mol. Cell* **68**, 515-527.e516 (2017). https://doi.org:10.1016/j.molcel.2017.10.014

8 Schmidt, A. *et al.* The quantitative and condition-dependent *Escherichia coli* proteome. *Nat. Biotechnol.* **34**, 104-110 (2016). https://doi.org:10.1038/nbt.3418
