# Supplementary material for: EF-P and its paralog EfpL (YeiP) differentially control translation of proline-containing sequences
Source: Nat Commun. 2024 Dec 2;15:10465. doi: 10.1038/s41467-024-54556-9 (PMC11611912; doi:10.1038/s41467-024-54556-9)
Supplement: Supplementary file 3 — Description of additional supplementary files [file 41467_2024_54556_MOESM3_ESM.pdf]

Description of Additional Supplementary Files

Supplementary Data 1: List of EF-P homologs
List of 5448 EF-P homologs extracted from a collection of 4763 complete bacterial genomes from the RefDeq database[1]. And a list of EfpL proteins and co-occuring EF-P paralogs from the total 5448 EF-P homologs.

Supplementary Data 2: Diffraction data collection and statistics of HADDOCK runs
Diffraction data collection and refinement statistics of Xray model and statistics of HADDOCK runs for docking and modelling of EF-P and EfpL complexes.

Supplementary Data 3: RiboSeq analysis on motifs and genes
Frequency of the motif to occur in pause site for *E. coli* BW25113, BW25113Δ*efp* and BW25113Δ*efp* +EfpL. Categorization to EF-P dependent or EfpL dependent according to differences in strains. Occurence of motif to be found in the whole *E. coli* BW25113 sequence. Asymmetry scores[2] of top 29 stalling motifs in different genes for *E. coli* BW25113, BW25113Δ*efp*, BW25113Δ*efpL* and BW25113Δ*efp* +EfpL. Categorization to EF-P dependent, EfpL dependent or dependent on both factors according to differences in strains. GO terms[3,4] categorized genes dependent on EF-P or EfpL.

Supplementary Data 4: Strains, Plasmids and Oligonucleotides
Strains, Plasmids and Oligonucleotides generated or used in this study with genotypes, decriptions and sequences.

Supplementary Data 5: Multiple Sequence Alignement
Sequences of "EFP_N" domains from these proteins were multiply aligned using Clustal Omega v.1.2.4[5] with all default parameters, shown in a multiple sequence alignment (MSA1). Clustal Omega v.1.2.4[5] with all default parameters was used to multiply align the sequences of KOW-like domains of the EfpL and EF-P proteins from the EfpL-containing genomes (MSA2).

Supplementary Data 6: Phylogenetic tree
Phylogenetic tree 1 (Supplementary Figure 1) and tree 2 (Fig. 1A, Supplementary Fig. 2) in Newick format, including bootstrap values.
