# Supplementary material for: EF-P and its paralog EfpL (YeiP) differentially control translation of proline-containing sequences
Source: Nat Commun. 2024 Dec 2;15:10465. doi: 10.1038/s41467-024-54556-9 (PMC11611912; doi:10.1038/s41467-024-54556-9)
Supplement: Supplementary file 10 — Reporting Summary [file 41467_2024_54556_MOESM10_ESM.pdf]

nature portfolio

Corresponding author(s): Jürgen Lassak

Last updated by author(s): Oct 8, 2024

Reporting Summary

Nature Portfolio wishes to improve the reproducibility of the work that we publish. This form provides structure for consistency and transparency in reporting. For further information on Nature Portfolio policies, see our Editorial Policies and the Editorial Policy Checklist.

Statistics

For all statistical analyses, confirm that the following items are present in the figure legend, table legend, main text, or Methods section.

n/a	Confirmed	
☐	☒	The exact sample size (*n*) for each experimental group/condition, given as a discrete number and unit of measurement
☐	☒	A statement on whether measurements were taken from distinct samples or whether the same sample was measured repeatedly
☐	☒	The statistical test(s) used AND whether they are one- or two-sided<br>*Only common tests should be described solely by name; describe more complex techniques in the Methods section.*
☒	☐	A description of all covariates tested
☒	☐	A description of any assumptions or corrections, such as tests of normality and adjustment for multiple comparisons
☐	☒	A full description of the statistical parameters including central tendency (e.g. means) or other basic estimates (e.g. regression coefficient) AND variation (e.g. standard deviation) or associated estimates of uncertainty (e.g. confidence intervals)
☐	☒	For null hypothesis testing, the test statistic (e.g. F, t, r) with confidence intervals, effect sizes, degrees of freedom and P value noted<br>*Give P values as exact values whenever suitable.*
☒	☐	For Bayesian analysis, information on the choice of priors and Markov chain Monte Carlo settings
☒	☐	For hierarchical and complex designs, identification of the appropriate level for tests and full reporting of outcomes
☒	☐	Estimates of effect sizes (e.g. Cohen's *d*, Pearson's *r*), indicating how they were calculated

Our web collection on statistics for biologists contains articles on many of the points above.

Software and code

Policy information about availability of computer code

Data collection	Reporter assays and growth measurements: CLARIOstar® PLUS, Tecan Infinity® or Tecan Infinite F500 microplate reader<br>Western blot imaging: Odyssey® CLx Imaging System (LI-COR, Inc)<br>Protein purification: Constant Systems Ltd. (Daventry, UK), ÄKTA pureTM chromatography system with Superdex 75 10/300 Increase column (Cytiva) and Resource Q (Bio-Rad).<br>Sequencing: Illumina's Nova-seq 6000 platform in 150PE mode<br>Phylogenetic tree: FastTree 2, R Version 4.3.2, ggplot2 version 3.4.4, IQ-TREE106 v1.6.12, IQ-TRÈE2.0.7<br>Structure determination: C3 ShotGun (SG1) crystallization screen (Molecular Dimension) in 96-well SWISSCI plates, EMBL P13 beamlines at the PETRA III storage ring of the DESY synchrotron<br>MS: Orbitrap Eclipse Tribrid Mass Spectrometer (Thermo Fisher Scientific), HESI-Spray source (Thermo Fisher Scientific), FAIMS interface (Thermo Fisher Scientific)<br>Databases: Integrated Microbial Genomes (IMG) database, Genome Taxonomy Database, RefSeq, Protein Database (PDB),
Data analysis	Data representation: GraphPad Prism Version 10.0.2 and 10.2.0, Affinity Designer2 2.2.1<br>Statistical analysis: GraphPad Prism Version 10.0.2 and 10.2.0, R Version 4.3.2<br>Image analysis: ImageJ 1.53e<br>Chromatography analysis: UNICORN 7.2.0.1200 control software<br>Sequencing analysis: Cutadapt, Python, Bowtie version 1.2, PausePred, HMMER v.3.4<br>Multiple Sequence Analysis: Clustal Omega v.1.2.4, MUSCLE v3.8.1551<br>Sequence logos: Weblogo<br>Structure analysis: autoproc+STARANISO, CCP4cloud, AlphaFold2, ModelCraft, PDB-REDO, REFMAC5, Coot, MolProbity server, PyMOL version 2.55 (Delano Scientific)

Docking and modelling: PyMol version 2.55 (Delano Scientific), HADDOCK
MS analysis: Freestyle 1.8 SP2 (Thermo Fisher Scientific) with Xtract Deconvolution algorithm
Genome/Proteome analysis: CheckM v1.0.12, GTDB-Tool kit (GTDB-Tk) version 0.2.2
Doubling time analysis: R package gRodon version 1.8.0,
Phylogenetic analysis: R package phytools version 2.0.3
Protein class enrichment analysis: PANTHER classification system (v.14.0)

For manuscripts utilizing custom algorithms or software that are central to the research but not yet described in published literature, software must be made available to editors and reviewers. We strongly encourage code deposition in a community repository (e.g. GitHub). See the Nature Portfolio guidelines for submitting code & software for further information.

Data

Policy information about availability of data

All manuscripts must include a data availability statement. This statement should provide the following information, where applicable:

- Accession codes, unique identifiers, or web links for publicly available datasets
- A description of any restrictions on data availability
- For clinical datasets or third party data, please ensure that the statement adheres to our policy

Source data are provided with this paper, including the relevant raw data, statistical test details and uncropped blots and pictures. The crystal structure of E. coli EfpL generated in this study have been deposited in the PDB database under accession code 8S8U. The structure of E. coli EF-P from Huter et al. [https://doi.org/10.1016/j.molcel.2017.10.014] was taken from the PDB database under accession code 6ENU [https://www.rcsb.org/structure/6ENU]. The ribosome profiling data generated in this study is available at SRD ID PRJNA1092679 [https://www.ncbi.nlm.nih.gov/bioproject/PRJNA1092679]. Data on the acylation status of EfpL under the tested conditions can be found in the following publications by Kuhn et al. [https://doi.org/10.1371/journal.pone.0094816], Weinert et al. [https://doi.org/10.1016/j.molcel.2013.06.003], Weinert et al. [https://doi.org/10.1016/j.celrep.2013.07.024] and Qian et al. [https://doi.org/10.1021/acs.jproteome.6b00264]. Quantitative E. coli proteome analysis data of Schmidt et al. [https://doi.org:10.1038/nbt.3418] was used to compare protein concentrations in different conditions.

Research involving human participants, their data, or biological material

Policy information about studies with human participants or human data. See also policy information about sex, gender (identity/presentation), and sexual orientation and race, ethnicity and racism.

Reporting on sex and gender	N/A
Reporting on race, ethnicity, or other socially relevant groupings	N/A
Population characteristics	N/A
Recruitment	N/A
Ethics oversight	N/A

Note that full information on the approval of the study protocol must also be provided in the manuscript.

Field-specific reporting

Please select the one below that is the best fit for your research. If you are not sure, read the appropriate sections before making your selection.

☒ Life sciences ☐ Behavioural & social sciences ☐ Ecological, evolutionary & environmental sciences

For a reference copy of the document with all sections, see nature.com/documents/nr-reporting-summary-flat.pdf

Life sciences study design

All studies must disclose on these points even when the disclosure is negative.

Sample size	Sample size was chosen based on the reproducibility of the data with statistically significant mean and standard deviation or determined based on the limitations of each method (E.g. Availability of genome and proteome datasets). At least 3 replicates were carried out for the analysis of cell-based assays. Sample size and replicate numbers are indicated where appropriate. The P-values are reported in the respective figure legends and the Source Data file.
Data exclusions	Predicted growth rates: We used CheckM v1.0.12 to assess the quality of each genome and retained only those that were predicted to be at least 90 % complete and contain less than 5 % contamination. We re-assigned taxonomy using the Genome Taxonomy Database and GTDB-Tool kit (GTDB-Tk) version 0.2.2 and removed genomes where the user-reported species did not agree with GTDB (removed 2 genomes). For example, we removed a genome with a user-reported species of Serratia marcescens 1822 which was sorted to the genus Rouxiella by GTDB-Tk. We also removed 14 genomes of endosymbionts from consideration, mainly from the genus Buchnera. We further subset for only those genomes which contained both genes for epmA and epmB (removed 62 genomes), contained at least one efp gene (removed 2 genomes) and

	had predicted doubling times under 24 hours (removed 15 genomes). This left 786 genomes for our analysis.
Replication	The number of biological/technical replicates are indicated in figure legends. Consistent results were obtained across replicates
Randomization	No randomization was performed in our study as appropriate control samples and defined conditions were used in our experiments
Blinding	Not relevant to our study due to the nature of the study

Reporting for specific materials, systems and methods

We require information from authors about some types of materials, experimental systems and methods used in many studies. Here, indicate whether each material, system or method listed is relevant to your study. If you are not sure if a list item applies to your research, read the appropriate section before selecting a response.

Materials & experimental systems

n/a	Involved in the study
☐	☒ Antibodies
☒	☐ Eukaryotic cell lines
☒	☐ Palaeontology and archaeology
☒	☐ Animals and other organisms
☒	☐ Clinical data
☒	☐ Dual use research of concern
☒	☐ Plants

Methods

n/a	Involved in the study
☒	☐ ChIP-seq
☒	☐ Flow cytometry
☒	☐ MRI-based neuroimaging

Antibodies

Antibodies used

1. 0.1 µg/ml rabbit monoclonal anti-6×His® antibody [RM146] (Abcam ab200537) as primary antibody for western blots
2. 0.2 µg/ml anti-rabbit IgG (H&L) [goat] alkaline phosphatase-conjugated (Rockland Immunochemicals 611-1502) as secondary antibody for western blots
3. 0.1 µg/ml donkey Anti-Rabbit IgG H&L (Alexa Fluor® 680) (Abcam ab175772) as secondary antibody for western blots
4. 0.1 µg/ml Anti-Acetylated-Lysine antibody, Rabbit monoclonal (Sigma-Aldrich SAB5600275) primary antibody for western blots

Validation

1. The manufacturers tested the anti-6×His® antibody [RM146] with 293T cells transfected with a His-tag fusion protein and E. coli lysate with His-tag protein and produced validation via images of western blots.
Reference:
Zouhir S, Abidi W, Caleechurn M, Krasteva PV. Structure and Multitasking of the c-di-GMP-Sensing Cellulose Secretion Regulator BcsE. mBio. 2020 Aug 11;11(4):e01303-20. doi: 10.1128/mBio.01303-20. PMID: 32788377; PMCID: PMC7439463.
2. The manufacturers tested the anti-rabbit IgG (H&L) [goat] alkaline phosphatase-conjugated antibody with 100ng Rabbit IgG and produced validation via images of western blots. Reaction visualized using alkaline phosphatase substrate for 30 seconds at RT.
References:
Guevara-Pantoja PE et al. Micro–nanoparticles magnetic trap: Toward high sensitivity and rapid microfluidic continuous flow enzyme immunoassay. Biomicrofluidics. (2020); Mueller A et al. In vitro assembly of Tobacco mosaic virus coat protein variants derived from fission yeast expression clones or plants. J Virol Methods. (2010);
Copello JA et al. Lack of effect of cADP-ribose and NAADP on the activity of skeletal muscle and heart ryanodine receptors. Cell Calcium. (2001); DeLamatre JG et al. Influence of dietary fat on the effect of endotoxin on murine hepatic peroxisomes. Hepatology. (1996)
3. The manufacturers tested the donkey Anti-Rabbit IgG H&L (Alexa Fluor® 680) against Anti-alpha Tubulin antibody - Microtubule Marker (AB18251) at 1 µg/mL and produced validation via images of western blots. Antibody binding was imaged using the Licor Odyssey CLx.
References:
Liu X, Reitsma JM, Mamrosh JL, Zhang Y, Straube R, Deshaies RJ. Cand1-Mediated Adaptive Exchange Mechanism Enables Variation in F-Box Protein Expression. Mol Cell. 2018 Mar 1;69(5):773-786.e6. doi: 10.1016/j.molcel.2018.01.038. PMID: 29499133; PMCID: PMC5836512;
Fefilova A, Melnikov P, Prikazchikova T, Abakumova T, Kurochkin I, Mazin PV, Ziganshin R, Sergeeva O, Zatsepin TS. Murine Long Noncoding RNA Morrbid Contributes in the Regulation of NRAS Splicing in Hepatocytes In Vitro. Int J Mol Sci. 2020 Aug 5;21(16):5605. doi: 10.3390/ijms21165605. PMID: 32764370; PMCID: PMC7460575;
Gao Y, Liu JF, Zhang C, Liu L, Liu YP, Zhang SL, Zhao LM. Enzyme-injected method of enzymatic dispersion at low temperature is effective for isolation of smooth muscle cells from human esophagogastric junction. Exp Ther Med. 2020 Apr;19(4):2933-2948. doi: 10.3892/etm.2020.8560. Epub 2020 Feb 26. PMID: 32256779; PMCID: PMC7086163;
Li X, Guo S, Xu T, He X, Sun Y, Chen X, Cao S, Si X, Liao W, Liao Y, Han Y, Bin J. Therapeutic ultrasound combined with microbubbles improves atherosclerotic plaque stability by selectively destroying the intraplaque neovasculature. Theranostics. 2020 Jan 22;10(6):2522-2537. doi: 10.7150/thno.39553. Erratum in: Theranostics. 2023 Apr 17;13(7):2259-2262. PMID: 32194817; PMCID: PMC7052908;
Behr M, Faleri C, Hausman JF, Planchon S, Renaut J, Cai G, Guerriero G. Distribution of cell-wall polysaccharides and proteins during growth of the hemp hypocotyl. Planta. 2019 Nov;250(5):1539-1556. doi: 10.1007/s00425-019-03245-9. Epub 2019 Jul 27. PMID: 31352512;
Taylor BK, Sinha GP, Donahue RR, Grachen CM, Morón JA, Doolen S. Opioid receptors inhibit the spinal AMPA receptor Ca2+ permeability that mediates latent pain sensitization. Exp Neurol. 2019 Apr;314:58-66. doi: 10.1016/j.expneurol.2019.01.003. Epub

2019 Jan 17. PMID: 30660616; PMCID: PMC6559354;
Li X, Sun Y, Huang S, Chen Y, Chen X, Li M, Si X, He X, Zheng H, Zhong L, Yang Y, Liao W, Liao Y, Chen G, Bin J. Inhibition of AZIN2-sv induces neovascularization and improves prognosis after myocardial infarction by blocking ubiquitin-dependent talin1 degradation and activating the Akt pathway. EBioMedicine. 2019 Jan;39:69-82. doi: 10.1016/j.ebiom.2018.12.001. Epub 2018 Dec 10. PMID: 30545799; PMCID: PMC6355659;
Zhang M, Jin X, Yang YF. β-Glucan from Saccharomyces cerevisiae induces SBD-1 production in ovine ruminal epithelial cells via the Dectin-1-Syk-NF-κB signaling pathway. Cell Signal. 2019 Jan;53:304-315. doi: 10.1016/j.cellsig.2018.10.018. Epub 2018 Oct 26. PMID: 30401641
4. The manufacturers the Anti-Acetylated-Lysine antibody on a western blot of HeLa cells nontreated or treated with Trichostatin A, and tested the antibody in multiplex recognizing acetylated lysine in peptides with different sequences. No cross reactivity with nonacetylated lysine, and lysine with other modification was detected.

Plants

Seed stocks	N/A
Novel plant genotypes	N/A
Authentication	N/A
